# Limits to the scalability of cortical network models

**DOI:** 10.1186/1471-2202-16-S1-O1

**Published:** 2015-12-18

**Authors:** Sacha J van Albada, Moritz Helias, Markus Diesmann

**Affiliations:** 1 Institute of Neuroscience and Medicine (INM-6) and Institute for Advanced Simulation (IAS-6), Jülich Research Centre and JARA, Jülich, Germany; 2 Department of Psychiatry, Psychotherapy and Psychosomatics, Medical Faculty, RWTH Aachen University, Aachen, Germany; 3 Department of Physics, Faculty 1, RWTH Aachen University, Aachen, Germany

## 

The size of the mammalian brain is inconveniently right in the middle between a few interacting particles and a mole of matter on a logarithmic scale. In physics, we learn that often in the limit where system size goes to infinity, simple mathematical expressions can be obtained uncovering the mechanisms governing the dynamics at the large but finite system size in nature. In neuroscience, however, we found that such an ansatz may fail because correlations drop so slowly that the mechanism governing the behavior in the infinite size limit [1] is not the mechanism relevant at the scale of the brain circuit in question [2]. The direct simulation of networks at their natural size has historically been difficult due to the sheer number of neurons and synapses. Therefore, neuroscientists also routinely explore the other side of the logarithmic scale and investigate downscaled circuits. In summary, it seems that brain networks are often too small for the infinity limit and too large for simulations.

In this contribution, we assess the scalability of networks in the asynchronous irregular state with a focus on downscaling. By extending the theory of correlations in such networks [2-5] and verifying analytical predictions by direct simulations using NEST [6], we formally demonstrate that generally already second-order measures cannot be preserved. The underlying mathematical reason is a one-to-one mapping between correlation structure and effective connectivity, which depends both on the physical connectivity and on the working point of the neurons [7]. Correlations are relevant because they influence synaptic plasticity [8] and large-scale measurements of neuronal activity [9], and are related to information processing and behavior [10,11].

Our results show that the reducibility of asynchronous networks is fundamentally limited, indicating the importance of considering networks with realistic numbers of neurons and synapses. Fortunately, corresponding simulation technology is becoming available to neuroscience [12]. Both the investigation of the infinity limit and the exploration of downscaled networks remain powerful methods of computational neuroscience. However, researchers should make explicit the rationale they apply in up- or downscaling.
